# Human bocavirus, coronavirus, and polyomavirus detected among patients hospitalised with severe acute respiratory illness in South Africa, 2012 to 2013

**DOI:** 10.1002/hsr2.59

**Published:** 2018-07-18

**Authors:** Kathleen Subramoney, Orienka Hellferscee, Marthi Pretorius, Stefano Tempia, Meredith McMorrow, Anne von Gottberg, Nicole Wolter, Ebrahim Variava, Halima Dawood, Kathleen Kahn, Sibongile Walaza, Shabir A. Madhi, Cheryl Cohen, Marietjie Venter, Florette K. Treurnicht

**Affiliations:** ^1^ National Institute for Communicable Diseases of the National Health Laboratory Service Centre for Respiratory Diseases and Meningitis Johannesburg South Africa; ^2^ University of the Witwatersrand School of Pathology, Faculty of Health Sciences Johannesburg South Africa; ^3^ Technical Research and Development, Novartis Pharma AG Basel Switzerland; ^4^ Centers for Disease Control and Prevention Influenza Division Atlanta Georgia USA; ^5^ Centers for Disease Control and Prevention Influenza Program Pretoria South Africa; ^6^ Klerksdorp‐Tshepong Hospital Complex Department of Medicine Klerksdorp South Africa; ^7^ University of the Witwatersrand Perinatal HIV Research Unit Johannesburg South Africa; ^8^ Pietermaritzburg Metropolitan Hospital Department of Medicine Pietermaritzburg South Africa; ^9^ University of KwaZulu‐Natal Department of Medicine Pietermaritzburg South Africa; ^10^ University of the Witwatersrand Medical Research Council, Respiratory and Meningeal Pathogens Research Unit Johannesburg South Africa; ^11^ University of the Witwatersrand Department of Science and Technology/National Research Foundation: Vaccine Preventable Diseases Johannesburg South Africa; ^12^ University of the Witwatersrand School of Public Health, Faculty of Health Sciences Johannesburg South Africa; ^13^ University of Pretoria Emerging Arbo And Respiratory Virus Program, Centre for Viral Zoonoses, Department of Medical Virology Pretoria South Africa

**Keywords:** human bocavirus, human coronavirus, human polyomavirus, respiratory tract infections, South Africa

## Abstract

**Aim:**

To investigate the prevalence of human bocavirus (hBoV), human coronaviruses (hCoV), and human polyomaviruses (hPyV) among patients with severe acute respiratory illness (SARI), in South Africa.

**Methods:**

The study included 680 South African patients randomly selected in age‐defined categories from hospitalised patients enrolled through SARI surveillance during 2012 to 2013. A multiplex reverse transcription real‐time polymerase chain reaction assay was used to detect hBoV; hCoV‐OC43, hCoV‐229E, hCoV‐NL63, and hCoV‐HKU1; and Washington University hPyV (hPyV‐WU) and Karolinska Insitute hPyV (hPyV‐KI), in respiratory tract specimens collected from patients with SARI. All respiratory specimens from patients enrolled through SARI surveillance were also routinely tested by multiplex reverse transcription real‐time polymerase chain reaction for adenovirus; enterovirus; human metapneumovirus; parainfluenza virus types 1, 2, and 3; respiratory syncytial virus; rhinovirus; influenza A, and influenza B.

**Results:**

Human bocavirus, hCoV‐229E, and hPyV‐WU were detected in 3.7% (25/680), 4.1% (28/680), and 4.1% (28/680) of respiratory specimens, respectively. All other viruses were detected in <2% of specimens. Rhinovirus was the most common coinfecting virus (21.4%‐60.7%), followed by adenovirus (21.4%‐39.3%), and respiratory syncytial virus (10.7%‐24.0%). Testing for the additional viruses (hBoV, hCoV, and hPyV) decreased the number of specimens that initially tested negative by 2.9% (20/680).

**Conclusion:**

Inclusion of laboratory tests for hBoV, hCoV‐229E, and hPyV‐WU in differential testing algorithms for surveillance and diagnostics for suspected cases of respiratory illness of unknown cause may improve our understanding of the etiology of SARI, especially in a country like South Africa with a high number of immune compromised persons.

## Abstract 2

Aim

To investigate the prevalence of human bocavirus (hBoV), human coronaviruses (hCoV), and human polyomaviruses (hPyV) among patients with severe acute respiratory illness (SARI), in South Africa.

Methods

The study included 680 South African patients randomly selected in age‐defined categories from hospitalised patients enrolled through SARI surveillance during 2012 to 2013. A multiplex reverse transcription real‐time polymerase chain reaction assay was used to detect hBoV; hCoV‐OC43, hCoV‐229E, hCoV‐NL63, and hCoV‐HKU1; and Washington University hPyV (hPyV‐WU) and Karolinska Insitute hPyV (hPyV‐KI), in respiratory tract specimens collected from patients with SARI. All respiratory specimens from patients enrolled through SARI surveillance were also routinely tested by multiplex reverse transcription real‐time polymerase chain reaction for adenovirus; enterovirus; human metapneumovirus; parainfluenza virus types 1, 2, and 3; respiratory syncytial virus; rhinovirus; influenza A, and influenza B.

Results

Human bocavirus, hCoV‐229E, and hPyV‐WU were detected in 3.7% (25/680), 4.1% (28/680), and 4.1% (28/680) of respiratory specimens, respectively. All other viruses were detected in <2% of specimens. Rhinovirus was the most common coinfecting virus (21.4%‐60.7%), followed by adenovirus (21.4%‐39.3%), and respiratory syncytial virus (10.7%‐24.0%). Testing for the additional viruses (hBoV, hCoV, and hPyV) decreased the number of specimens that initially tested negative by 2.9% (20/680).

Conclusion

Inclusion of laboratory tests for hBoV, hCoV‐229E, and hPyV‐WU in differential testing algorithms for surveillance and diagnostics for suspected cases of respiratory illness of unknown cause may improve our understanding of the etiology of SARI, especially in a country like South Africa with a high number of immune compromised persons.

## 1 INTRODUCTION

Acute respiratory tract infections cause substantial mortality and morbidity in all ages globally^1^. Apart from influenza and respiratory syncytial viruses (RSVs), several other respiratory viruses remain largely undiagnosed^1^. Understanding and defining the contribution of viral etiologies of severe respiratory illness in countries like South Africa that have a high burden of human immunodeficiency virus (HIV) infections and associated acquired immunodeficiency syndrome may be important for health management and planning. In a prior South African study, 43% of respiratory specimens from patients hospitalised with severe acute respiratory illness (SARI) tested negative for 10 screened viruses (parainfluenza virus [PIV] types 1, 2 and 3; RSV; enterovirus; human metapneumovirus [HMPV]; adenovirus [AdV]; rhinovirus [RV]; influenza A [INFA]; and influenza B [INFB])^2^. Respiratory viruses not included in routine diagnostic testing such as human bocavirus (hBoV), human coronaviruses (hCoVs), and human polyomaviruses (hPyV) may be responsible for viral respiratory illness among patients that test negative for the more commonly screened viruses.

Human bocavirus, hPyV, and hCoV infections have been reported among patients with respiratory tract infections in different settings^3^, ^4^, ^5^, ^6^, but these viruses are usually poorly investigated in routine surveillance or for diagnostic purposes. We aimed to assess the prevalence of hBoV, hCoV‐OC43, hCoVs‐229E, hCoVs‐NL63, hCoVs‐HKU1, Washington University human polyomavirus (hPyV‐WU), and Karolinska Insitute human polyomavirus (hPyV‐KI) among hospitalised patients with SARI in South Africa, during 2012 to 2013.

## 2 METHODS

### 2.1 Study population

Study participants were enrolled in prospective, hospital‐based SARI surveillance, from January 2012 through December 2013. Severe acute respiratory illness surveillance was conducted in 5 sentinel sites in North West (Klerksdorp‐Tshepong Hospital Complex, Klerksdorp), KwaZulu‐Natal (Edendale Hospital, Pietermaritzburg), Mpumalanga (Matikwana and Mapulaneng Hospitals, Agincourt), and Gauteng (Chris Hani Baragwanath Hospital) provinces in South Africa. Severe acute respiratory illness cases were defined as hospitalised patients with symptom onset within 7 days (symptom onset duration changed to ≤10 days from 2013) of hospital admission and who met age‐specific inclusion criteria, including (1) children aged 2 days to <3 months with physician‐diagnosed/suspected sepsis or acute lower respiratory tract infection, irrespective of signs and symptoms; (2) children aged 3 months to <5 years with physician‐diagnosed acute lower respiratory tract infection or pleural effusion; and (3) individuals aged ≥5 years with physician diagnosed lower respiratory tract infection with sudden onset of temperature ≥38°C or history of fever, cough or sore throat, shortness of breath, or difficulty breathing.^7^, ^8^

### 2.2 Study procedures

Study procedures have been described previously.^9^, ^10^ Briefly, study staff reviewed hospital admission registers to identify patients meeting the SARI case definition. All patients that met the case definition were approached and consented by the surveillance officers. Patients who consented to participate in the study were asked to complete a questionnaire by interview to record previous medical history and clinical data. Consented patients were asked to provide respiratory and other clinical specimens; at the time, permissions were obtained to use stored specimens for research studies.

### 2.3 Determination of HIV status

For consenting patients, HIV results were obtained from medical records available at the time of enrollment, or testing of dried‐blood spots were performed at the National Institute for Communicable Diseases, Johannesburg, South Africa. Human immunodeficiency virus testing included polymerase chain reaction (PCR)for children aged <18 months and enzyme‐linked immunosorbent assay for patients aged ≥18 months.

### 2.4 Study specimens, sample size, and laboratory procedures

Nasopharyngeal aspirates were collected from children aged <5 years, and combined nasopharyngeal and oropharyngeal swabs were collected from persons aged ≥5 years. Specimens were placed in viral (Highveld Biological, Johannesburg, South Africa) or universal (Copan, Murrieta, California, USA) transport medium and stored at 2 to 8°C. Within 72 hours of collection, the specimens were transported to the National Institute for Communicable Diseases (Johannesburg, South Africa) for testing. Respiratory specimens were stored at −70°C.

After sorting by collection date, a subset of respiratory specimens was randomly selected in age‐categories (<1, 1‐4, 5‐24, 25‐44, 45‐65, and > 65 years of age), for inclusion in this study. From a total of 5784 specimens collected during the surveillance period, every eighth specimen was then systematically selected for inclusion in this study to obtain a study sample of 680 (11.8%).

The specimens were tested using a multiplex reverse transcription real‐time PCR (RT‐qPCR) assay for the following viruses: hBoV, hCoV‐OC43, hCoV‐229E, hCoV‐NL63, hCoV‐HKU1, hPyV‐WU, and hPyV‐KI.^11^, ^12^ The RT‐qPCR assay was performed using parameters and cycling conditions previously described,^2^ with the following modifications: 2.27 μM anchored‐oligo (dT)_18_ primer and 54.55 μM random hexamer primer was used in the reverse transcription reaction. The respiratory specimens from SARI surveillance were also routinely tested by multiplex RT‐qPCR assay for AdV, enterovirus, HMPV, PIV types 1 to 3, RSV, RV, INFA, and INFB.^2^

### 2.5 Statistical analysis

Prevalence of hBoV, hCoV, and hPyV viruses were compared between age groups, HIV‐infected and HIV‐uninfected individuals, and individuals with and without non‐HIV underlying medical conditions (predominantly, asthma, heart disease, pregnancy, diabetes, seizure disorders, and malnutrition) using the chi‐square test or Fisher exact test. Stata® version 14 (StataCorp, College Station, Texas, USA) was used to implement the analysis.

### 2.6 Ethical approval

The protocol was approved by the University of the Witwatersrand (M081042) and the University of KwaZulu‐Natal (BF157/08) ethics committees. The surveillance programme was deemed nonresearch by the U.S. Centers for Disease Control and Prevention (nonresearch determination number: 2012‐6197).

## 3 RESULTS

### 3.1 Study population

Among the patients included in this study, children aged <1 year accounted for the largest proportion of cases (46.8%; 318/680). Among individuals with available information, the HIV prevalence was 30.5% (204/668), and the prevalence of non‐HIV underlying medical conditions was 7.5% (51/678). CD4^+^ count information was only available for a limited number of individuals; therefore, these results could not be utilized. Age distribution and HIV prevalence of cases included in and excluded from this study were statistically similar (Table 1).

**Table 1:** Distribution of patients hospitalised with severe acute respiratory illness tested for hBoV, hPyV, and hCoV viruses at 5 sentinel surveillance sites in South Africa, 2012 to 2013

Demographic and Clinical Characteristics	Not Tested n/N (%)	Tested n/N (%)	*P* value
All	5104/5784 (88.2)	680/5784 (11.8)	
Age, years			.012^b^
<1	2010/5104 (39.4)	318/680 (46.8)	
1 to 4	1149/5104 (22.5)	144/680 (21.2)	
5 to 24	340/5104 (6.7)	37/680 (5.4)	
25 to 44	1013/5104 (19.8)	117/680 (17.2)	
45 to 65	465/5104 (9.1)	50/680 (7.4)	
>65	127/5104 (2.5)	14/773 (1.8)	
HIV			.093^c^
Yes	1394/4115 (33.9)	204/668 (30.5)	
No	2721/4115 (66.1)	464/668 (69.5)	
Non‐HIV underlying medical conditions^a^			.276^d^
Yes	450/5096 (8.8)	51/678 (7.5)	
No	4646/5096 (91.2)	627/678 (92.5)	

Abbreviations: hBoV, human bocavirus; hCoV, human coronaviruses; HIV, human immunodeficiency virus; hPyV, human polyomaviruses.

a Asthma, other chronic lung disease, cerebrovascular accident/stroke, chronic renal failure, heart failure, valvular heart disease, coronary artery disease, immunosuppressive therapy, cortisone, chemotherapy, radiation therapy, organ transplant, pregnancy, sickle cell disease, splenectomy, diabetes, burns, immunoglobulin deficiency, autoimmune disease, kwashiorkor/marasmus, nephritic syndrome, spinal cord injury, seizure disorder, prematurity, obesity, chronic obstructive pulmonary disease/emphysema, malignancy/cancer, and malnutrition.

*P* values: determined using the chi‐square test

b *P* values comparing the distribution of patients among the various age groups between those tested and not tested for viruses.

c *P* values comparing the distribution of HIV‐infected and HIV‐uninfected patients between those tested and not tested for viruses.

d *P* values comparing the distribution of individuals with and without non‐HIV underlying medical conditions between those tested and not tested for viruses.

### 3.2 Prevalence of hBoV, hCoV, hPyV, and commonly tested viruses

Among the hBoV, hCoV, and hPyV, hBoV (3.7%; 25/680), hCoV‐229E (4.1%; 28/680), and hPyV‐WU (4.1%; 28/680) were the most frequently detected. Human coronaviruses‐OC43, hCoV‐NL63, hCoV‐HKU1, and hPyV‐KI were each detected in <2% of specimens (Table 2). The prevalence of hBoV, hCoV‐229E, and hPyV‐WU were similar or higher than those of some other respiratory viruses commonly tested for, including HMPV (3.5%; 24/680), PIV‐1 (2.1%; 14/680), PIV‐2 (0.9%; 6/680), PIV‐3 (3.5%; 24/680), INFA (2.8%; 19/680), and INFB (2.6%; 18/680) (Table 3).

**Table 2:** Prevalence of hBoV, hPyV, and hCoV viruses among patients hospitalised with severe acute respiratory illness at 5 sentinel surveillance sites in South Africa, 2012 to 2013

Demographic And Clinical characteristics	hBoV		hPyV‐WU		hPyV‐KI		hCoV‐OC43		hCoV‐229E		hCoV‐NL63		hCoV‐HKU1	
	n/N (%)	*P* value	n/N (%)	*P* value	n/N (%)	*P* value	n/N (%)	*P* value	n/N (%)	*P* value	n/N (%)	*P* value	n/N (%)	*P* value
All	25/680 (3.7)		28/680 (4.1)		13/680 (1.9)		2/680 (0.3)		28/680 (4.1)		2/680 (0.3)		1/680 (0.1)	
Age, years		.274^b^		0.001		.733		1.000		.239		‐		.532
<1	15/318 (4.7)		11/318 (3.5)		5/318 (1.6)		2/318 (0.6)		8/318 (2.5)		0/318 (0.0)		0/318 (0.0)	
1 to 4	8/144 (5.6)		16/144 (11.1)		4/144 (2.8)		0/144 (0.0)		9/144 (6.3)		0/144 (0.0)		1/144 (0.7)	
5 to 24	0/37 (0.0)		0/37 (0.0)		1/37 (2.7)		0/37 (0.0)		1/37 (2.7)		0/37 (0.0)		0/37 (0.0)	
25 to 44	2/117 (1.7)		1/117 (0.9)		3/117 (2.6)		0/117 (0.0)		7/117 (6.0)		2/117 (1.7)		0/117 (0.0)	
45 to 65	0/50 (0.0)		0/50 (0.0)		0/50 (0.0)		0/50 (0.0)		2/50 (4.0)		0/50 (0.0)		0/50 (0.0)	
>65	0/14 (0.0)		0/14 (0.0)		0/14 (0.0)		0/14 (0.0)		1/14 (7.1)		0/14 (0.0)		0/14 (0.0)	
HIV		.656^c^		1.000		.074		1.000		.286		‐		1.000
Yes	6/204 (2.9)		8/204 (3.9)		7/204 (3.4)		0/204 (0.0)		11/204 (5.4)		0/204 (0.0)		0/204 (0.0)	
No	18/464 (3.9)		19/464 (4.1)		6/464 (1.3)		2/464 (0.4)		16/464 (3.4)		0/464 (0.0)		1/464 (0.2)	
Non‐HIV underlying medical conditions^a^		1.000^d^		.714		.255		1.000		.713		‐		.075
Yes	1/51 (2.0)		1/51 (2.0)		2/51 (3.9)		0/51 (0.0)		1/51 (2.0)		0/51 (0.0)		1/51 (2.0)	
No	24/627 (3.8)		27/627 (4.3)		11/627 (1.8)		2/627 (0.3)		26/627 (4.1)		0/627 (0.0)		0/627 (0.0)	

Abbreviations: hBoV, human bocavirus; hCoV‐NL63, human coronavirus NL63; hCoV‐HKU1, human coronavirus HKU1; hCoV‐OC43, human coronavirus OC43; hCoV‐229E, human coronavirus 229E; HIV, human immunodeficiency virus; hPyV‐KI, Karolinska Insitute human polyomavirus; hPyV‐WU, Washington University human polyomavirus.

a Asthma, other chronic lung disease, cerebrovascular accident/stroke, chronic renal failure, heart failure, valvular heart disease, coronary artery disease, immunosuppressive therapy, cortisone, chemotherapy, radiation therapy, organ transplant, pregnancy, sickle cell disease, splenectomy, diabetes, burns, immunoglobulin deficiency, autoimmune disease, kwashiorkor/marasmus, nephritic syndrome, spinal cord injury, seizure disorder, prematurity, obesity, chronic obstructive pulmonary disease/emphysema, malignancy/cancer, and malnutrition.

*P* values: determined using the Fisher's exact test and values <0.05 represents statistical significance within that category.

b *P* values comparing the prevalence of each virus between patients in the various age groups.

c *P* values comparing the prevalence of each virus between HIV‐infected and HIV‐uninfected patients.

d *P* values comparing the prevalence of each virus between individuals with and without non‐HIV underlying medical conditions.

–: *P* value could not be determined.

**Table 3:** Prevalence of commonly tested viruses among patients hospitalised with SARI at 5 sentinel in South Africa, 2012 to 2013

Demographic and Clinical characteristics	AdV		EV		HMPV	PIV‐1			PIV‐2		PIV‐3		RSV		RV		INFA		INFB	
	n/N (%)	*P* value	n/N (%)	*P* value	n/N (%)	*P* value	n/N (%)	*P* value	n/N (%)	*P* value	n/N (%)	*P* value	n/N (%)	*P* value	n/N (%)	*P* value	n/N (%)	*P* value	n/N (%)	*P* value
All	96/680 (14.1)		44/680 (6.5)		24/680 (3.5)		14/680 (2.1)		6/680 (0.9)		24/680 (3.5)		135/680 (19.9)		216/680 (31.8)		19/680 (2.8)		18/680 (2.6)	
Age, years		<.001^a^		<.001		.265		.549		.948		.126		<.001		<.001		.974		.016
<1	51/318 (16.0)		22/318 (6.9)		14/318 (4.4)		5/318 (1.6)		3/318 (0.9)		18/318 (5.7)		103/318 (32.4)		111/318 (34.9)		10/318 (3.1)		4/318 (1.3)	
1 to 4	36/144 (25.0)		18/144 (12.5)		7/144 (4.9)		6/144 (4.2)		2/144 (1.4)		3/144 (2.1)		22/144 (15.3)		60/144 (41.7)		4/144 (2.8)		3/144 (2.1)	
5 to 24	2/37 (5.4)		4/37 (10.8)		0/37 (0.0)		0/37 (0.0)		0/37 (0.0)		0/37 (0.0)		1/37 (2.7)		14/37 (37.8)		0/37 (0.0)		1/37 (2.7)	
25 to 44	4/117 (3.4)		0/117 (0.0)		2/117 (1.7)		2/117 (1.7)		1/117 (0.9)		1/117 (0.9)		4/117 (3.4)		21/117 (17.9)		4/117 (3.4)		4/117 (3.4)	
45 to 65	1/50 (2.0)		0/50 (0.0)		0/50 (0.0)		1/50 (2.0)		0/50 (0.0)		2/50 (4.0)		4/50 (8.0)		7/50 (14.0)		1/50 (2.0)		5/50 (10.0)	
>65	2/14 (14.3)		0/14 (0.0)		1/14 (7.1)		0/14 (0.00)		0/14 (0.0)		0/14 (0.0)		1/14 (7.1)		3/14 (21.4)		0/14 (0.0)		1/14 (7.1)	

Abbreviations: AdV, adenovirus; EV, enterovirus; HMPV, human metapneumonvirus; INFA, influenza virus A; INFB, influenza virus B; PIV‐1, parainfluenza virus 1; PIV‐2, parainfluenza virus 2; PIV‐3, parainfluenza virus 3; RSV, respiratory syncytial virus; RV, rhinovirus; SARI, severe acute respiratory illness.

*P* values: determined using the Fisher's exact test and values <0.05 represents statistical significance within that category.

a *P* values comparing the prevalence of each virus between patients in the various age groups.

Human bocavirus (5.6%; 8/144), hCoV‐229E (6.3%; 9/144), and hPyV‐WU (11.1%; 16/144), were the most frequently detected among children aged 1 to 4 years. A significant difference in the prevalence across age groups was only observed for hPyV‐WU (*P* = .001, Fisher's exact test). Among the commonly tested viruses in this age group, HMPV; PIV 1, 2, and 3; INFA; and INFB virus had a lower prevalence, of 4.9% (7/144), 4.2% (6/144), 1.4% (2/144), 2.1% (3/144), 2.8% (4/144), and 2.1% (3/144), respectively, compared with the hBoV, hCoV, and hPyV. In this study, 29.6% (201/680) of study specimens tested negative for viral targets from both RT‐qPCR assays and, therefore, testing for the additional viruses (hBoV, hCoV, and hPyV) decreased the number of specimens that initially tested negative by 2.9% (20/680).

Human bocavirus (2.9%; 6/204), hCoV‐229E (5.4%; 11/204), hPyV‐WU (3.9%; 8/204), and hPyV‐KI (3.4%; 7/204) were most commonly detected among HIV‐infected individuals. However, no statistical significance was observed when HIV‐infected individuals were compared with HIV‐uninfected individuals (Table 2). Karolinska Insitute human polyomavirus (3.9%; 2/51) was also most frequently detected among patients with non‐HIV underlying medical conditions; this difference, however, was not statistically significant (Table 2).

Twenty‐eight percent (7/25), 21.4% (6/28), and 60.7% (17/28) of patients infected with hBoV, hCoV‐229E, and hPyV‐WU were coinfected with other viruses, respectively. Among the coinfecting viruses, RV was the most common (21.4‐60.7%), followed by AdV (21.4‐39.3%) and RSV (10.7‐24.0%). Coinfections with INFA or INFB viruses were not observed.

## 4 DISCUSSION

The detection rate of hBoV, hCoV, and hPyV‐WU was <4.2%. Children aged 1 to 4 years were more likely to be infected with hBoV, hCoV‐229E, or hPyV‐WU in comparison with older children and adults. The overall prevalence of hBoV, hCoV‐229E, and hPyV‐WU among patients with SARI were similar or higher than those of some more commonly detected respiratory viruses such as HMPV, PIV types 1 to 3, INFA, and INFB. However, the high coinfection rate observed, especially among hPyV‐WU infected patients with other viruses, will make the interpretation of the etiological association difficult and should be kept in mind in studies that would aim to address this. Although we detected hBoV in 3.7% (25/680) of specimens, we did not test these cases for hBoV mRNA or viral DNA load to assess the proportion of cases with active infection as described by Xu et al.^13^

In a similar study conducted in South Africa during 2006 to 2007, 610 hospitalised children aged <5 years that required medical attention or hospitalization were tested for several respiratory viruses including those tested in our study^14^. Among these children, hBoV, hCoV‐229E, and hPyV‐WU were detected in 6.1% (37/610), 0.3% (2/610), and 3.4% (21/610) of specimens, respectively. In our study, a higher prevalence of hCoV‐229E was reported (Table 2). This may be due to the different case definitions used in the 2 studies. Findings similar to ours were reported in a study conducted in the Gulf region in children <5 years of age. In the latter study, the reported detection rates for these viruses were 4.9% (14/331) hBoV and hCoV‐229E, and 3.5% (10/331) hPyV‐WU.^5^ Globally, hBoV and hPyV‐WU are predominantly observed in children aged <4 years with upper and lower respiratory tract infection, which was also observed in our study.^15^ A recent South African study found that among children aged <1 year, hBoV (but not hCoV) detection was significantly associated with severe illness.^16^ Human polyomaviruses was not investigated in the latter study. Therefore, inclusion of laboratory tests for hBoV, hCoV‐229E, and hPyV‐WU in differential testing algorithms for surveillance and diagnostics for suspected cases of respiratory illness of unknown cause may improve our understanding of the etiology of SARI, especially in a country like South Africa with a high number of immune compromised persons.

This study has limitations that warrant discussion. First, only patients with SARI were included in this study. As no control participant group was evaluated, the association and/or fraction of hBoV, hCoV, and hPyV detections that may contribute to severe respiratory illness could not be investigated. Second, the sample size was too small. Sero‐prevalence data for South Africa is lacking; therefore, using 50% expected prevalence to obtain the biggest sample size for a 95% confidence interval, a 5% desired absolute precision, and a design effect of 1, plus inflation by 25% for spoilage/quality of samples, the annual expected sample for the study should have been 480 (960 for the 2 years). Lastly, the study duration was too short, as a 4‐ to 5‐year period is required to assess seasonality and, therefore, the seasonality of HBoV, hCoV, and hPyV was not determined.

In conclusion, while low, the prevalence of hBoV, hCoV‐229E, and hPyV‐WU were similar to or higher than some other important viruses commonly tested for such as HMPV, PIV types 1 to 3, INFA, and INFB. Including tests for hBoV, hCoV, and hPyV viruses in differential diagnostic algorithms for surveillance and routine diagnostics may improve our understanding of the viral etiology of SARI in hospitalised patients in a high HIV/AIDS burden country like South Africa. However, the association of these virus detections with severe respiratory illness should be investigated further. Presently, in South Africa, in order to maintain sustainability of our respiratory illness surveillance programme, expanded testing for viral etiologies are mainly conducted in patients with SARI (including deaths and intensive care unit admissions) of unknown cause through outbreak investigations and specialized diagnostic test requests, where identification of an etiologic agent is desired for possible public health action.

## FUNDING INFORMATION

This work was supported by the National Institute for Communicable Diseases of the National Health Laboratory Service and the Centers for Disease Control and Prevention (cooperative agreement no. 5U51IP000155).

## CONFLICT OF INTEREST

No conflict of interest was declared by any of the coauthors.

## AUTHOR CONTRIBUTIONS

Conceptualization: Marietjie Venter, Orienka Hellferscee, Marthi Pretorius, Cheryl Cohen, Florette K. Treurnicht

Data curation: Kathleen Subramoney, Orienka Hellferscee, Stefano Tempia, Meredith McMorrow, Anne von Gottberg

Formal analysis: Kathleen Subramoney, Orienka Hellferscee, Stefano Tempia, Meredith McMorrow, Anne von Gottberg

Investigation: Marietjie Venter, Orienka Hellferscee, Marthi Pretorius, Cheryl Cohen, Florette K. Treurnicht

Methodology: Orienka Hellferscee, Marthi Pretorius, Anne von Gottberg, Nicole Wolter, Ebrahim Variava, Halima Dawood, Kathleen Kahn, Sibongile Walaza, Cheryl Cohen, Marietjie Venter, Florette K. Treurnicht

Resources: Orienka Hellferscee, Marthi Pretorius, Anne von Gottberg, Nicole Wolter, Ebrahim Variava, Halima Dawood, Kathleen Kahn, Sibongile Walaza, Cheryl Cohen, Marietjie Venter, Florette K. Treurnicht

Software: Kathleen Subramoney, Orienka Hellferscee, Marthi Pretorius, Stefano Tempia, Meredith McMorrow, Nicole Wolter, Sibongile Walaza

Writing‐original draft preparation: Kathleen Subramoney, Orienka Hellferscee, Florette K. Treurnicht

Writing‐review and editing: Kathleen Subramoney, Orienka Hellferscee, Marthi Pretorius, Stefano Tempia Meredith McMorrow, Anne von Gottberg, Nicole Wolter, Sibongile Walaza, Shabir A. Madhi, Cheryl Cohen, Florette K. Treurnicht, Marietjie Venter

## DISCLAIMER

The findings and conclusions in this report are those of the authors and do not necessarily represent the official position of the Centers for Disease Control and Prevention.
